# Data on terrestrial ferns species richness, abundance, and functional traits in Mashpi Rainforest Biodiversity Reserve in the Ecuadorian Chocó

**DOI:** 10.1016/j.dib.2022.108784

**Published:** 2022-11-26

**Authors:** Laura Salazar, Paola Peña, Renato Segura, Mateo Roldán

**Affiliations:** a Universidad Tecnológica Indoamérica, Centro de Investigación de la Biodiversidad y Cambio Climático (BioCamb) y Facultad de Ciencias de Medio Ambiente - Ingeniería en Biodiversidad y Recursos Genéticos, Machala y Sabanilla, CP. EC170103, Quito, Ecuador.; b Universidad Regional Amazónica Ikiam. Km 7 vía Muyuna. Tena, Napo, Ecuador.; c Departamento de Investigación y Biología, Mashpi Lodge – Reserva Mashpi Reserve, Ecuador.

**Keywords:** Species richness, Species abundance, Chocó forest, Ferns, Leaf length, Leaf thickness

## Abstract

This data paper summarizes the data of a first survey of terrestrial ferns at Mashpi Biodiversity Reserve, an Ecuadorian Chocó forest relict, one of the most biodiverse areas in the world. We established 10 permanent plots of 400 m^2^ distributed in two elevational levels (800 and 1000 m a.s.l.) to register all species per plot and the abundance per species. In addition, we measured two morphological leaf functional traits of the species. We include a file with three tables, the first one includes a species list with scientific names and vouchers. The second one includes the abundance of each species per plot. The third one contains measurements of the leaf length and leaf thickness of several leaf samples of 28 species, representing the leaf functional traits of the species. This article also includes a table with coordinates and elevations of the plots and five figures with information about the number of genus and species per family, geographic location of plots and, the methodology for data collection. These data can be useful for plant ecologists to assess future changes of fern species composition and leaf functional traits of ferns caused by climate change and other threats at the study area.

**Specifications Table**

**Table:** 

Subject	Agricultural and Biological Sciences
Specific subject area	Plant Science
Type of data	Tables and Figures
How data were acquired	Field sampling measurements in 10 plots.
Data format	Raw Analyzed
Description of data collection	Data include species richness and abundance of all terrestrial ferns registered in the study plots. The data also include two morphological leaf functional traits of 28 species: the leaf length and leaf thickness.
Data source location	Mashpi Biodiversity Reserve, in Ecuador. Geographic location of the permanent sampling plots: Plot_1 0.16282 -78.88617 Plot-2 0.16362 -78.88596 Plot_3 0.16274 -78.8866 Plot_4 0.1645 -78.88598 Plot_5 0.16446 -78.88616 Plot_6 0.16493 -78.87395 Plot_7 0.1629 -78.87345 Plot_8 0.16526 -78.87338 Plot_9 0.16794 -78.87556 Plot_10 0.16804 -78.87531
Data accessibility	Data are available in this article and at Mendeley Data repository. Link to the dataset: https://data.mendeley.com/datasets/kn5f76dzwd/5 DOI: 10.17632/kn5f76dzwd.5

## Value of the Data

Our sample represents a first survey of species richness, abundance and leaf traits of terrestrial ferns at Mashpi Biodiversity Reserve, an Ecuadorian Chocó forest relict, representing one of the most biodiverse areas in the world.

• The data of leaf traits can provide insights on functional diversity of ferns at Mashpi Biodiversity Reserve.
• The data of leaf thickness can be used as indicators of water deficit.
• Plots of 400 m^2^ are commonly used in fern samples, so this dataset can be useful for pteridologists or ecologists to compare with other studies of terrestrial fern species richness.
• These data can be useful to assess future changes of fern species composition and leaf functional traits of ferns caused by climate change and other threats at the study area.

## 1 Objective

Most studies on tropical plant diversity focus on trees, the main structural component of forests. Although fully justified, such research ignores most tropical plants, which belong to the herbaceous and shrubby life forms [1]. For this reason, our research group focuses on evaluating the ecology of ferns along tropical elevational gradients. This data set shows the abundance, species richness and some leaf functional traits of one of the elevational ranges of our project.

## 2 Data Description

**Fig. 1:**
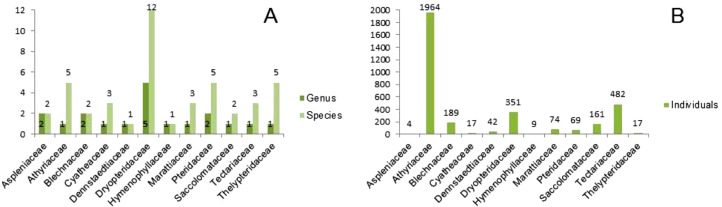
Number of genus and species per family (A) and total individual number per family (B).

**Fig. 2:**
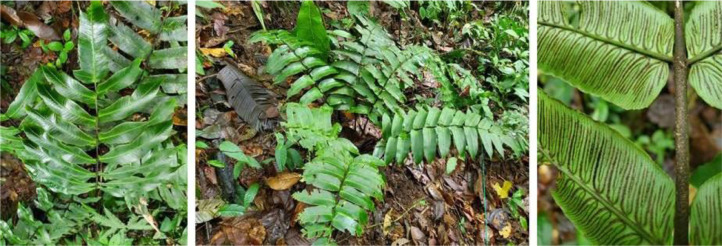
*Diplazium oellgaardii,* the most common species at the Mashpi Biodiversity Reserve.

This article reports the data of ten plots with reference to species richness, abundance (number of individuals) per species and plot and, the leaf length and leaf thickness of terrestrial ferns in the Mashpi Biodiversity Reserve. In total, we registered 3385 individuals of terrestrial fern, which are classified in 12 families, 19 genus and 47 terrestrial fern species detailed in Fig. 1 and file deposited at https://data.mendeley.com/datasets/kn5f76dzwd/5 [2]. Table “Species_vouchers” of the file details the family, scientific names, and voucher of each specie. Table “Community_matrix” contains the data to calculate the abundance of each species, the total abundance per plot, the species richness per plot and diversity index per plot. For this purpose, we also include another file with an R script. Fig. 1 displays the number of genus and species per family (A) and the total number of individuals (B) per family. Fig. 2 shows the most common species in the Mashpi Biodiversity Reserve.

The data described in Table “Functional_traits” from the file deposited at https://data.mendeley.com/datasets/kn5f76dzwd/5 [2] include the leaf length and leaf thickness of several leaf samples of 28 species representing the leaf functional traits of the species.

## 3 Experimental Design, Materials, and Methods

### 3.1 Study area

**Fig. 3:**
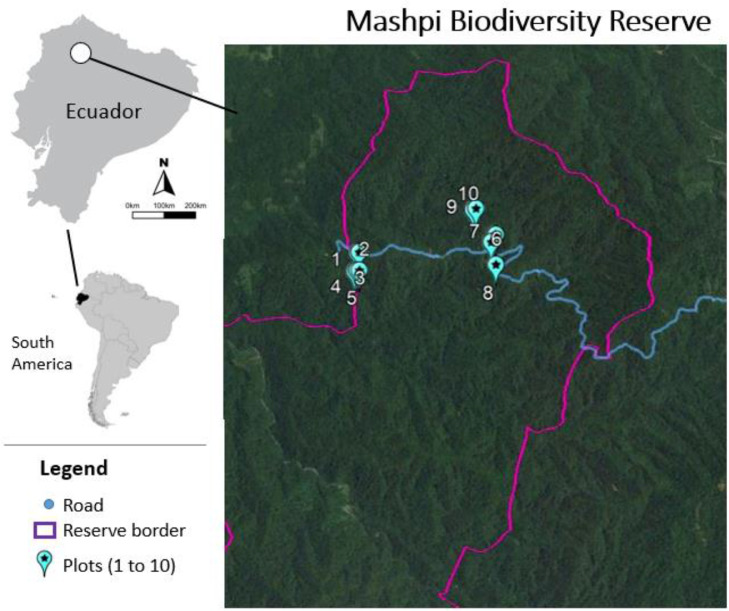
Map of the geographic location of the ten permanent plots of 400 m^2^ in the Mashpi Biodiversity Reserve.

Our survey was conducted in the Mashpi Biodiversity Reserve (Fig. 3), a private natural forest reserve in northwestern Ecuador. As a remnant of the Choco forests, this area represents one of the most biodiverse areas in the world [3]. The reserve was established in 2001 and nowadays expands to 2900 hectares. The area is characterized by rough and mountainous terrain (slopes between 45° to 90°) [4] and a mixed composition of primary forest (∼70%), secondary forest (∼25%) and forest on-going natural regeneration (∼5%). Within the reserve operates Mashpi Lodge (www.mashpilodge.com) [5] which has set-up a Research & Biology program that promotes and facilitates ecological research and conservation practices.

### 3.2 Data collection

**Fig. 4:**
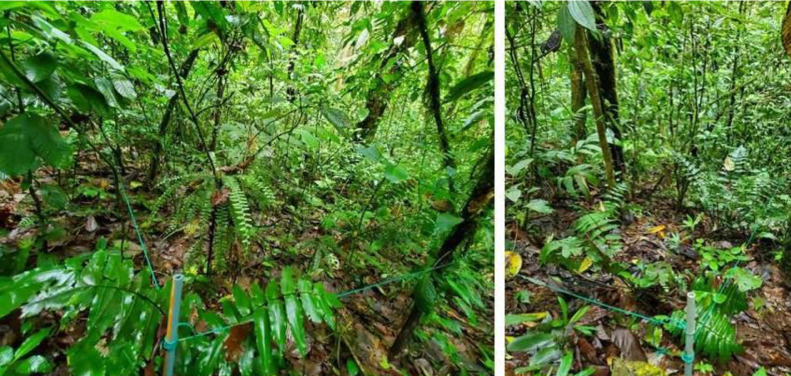
View of the corner of the plots marked with PVC tubes and delimited with green rope.

The data collection took place between August 2021 and May 2022. We established 10 permanent plots of 400 m^2^ (20 × 20 m) distributed in two elevational levels, five around 800 m a.s.l. and the other five around 1000 m a.s.l as shown in Fig. 3 and Table 1. The plots were marked with 1/4 inch PVC tubes in each corner and delimited with rope (Fig. 4).

**Table 1:** Coordinates and elevations of the ten permanent plots.

Plot	Coordinates		Elevation (m a.s.l.)	Species number	Total abundance
1	0.16282	-78.88617	807	13	242
2	0.16362	-78.88596	824	21	261
3	0.16274	-78.8866	806	21	275
4	0.1645	-78.88598	841	16	282
5	0.16446	-78.88616	844	14	303
6	0.16493	-78.87395	1013	21	451
7	0.1629	-78.87345	1023	8	220
8	0.16526	-78.87338	1008	22	573
9	0.16794	-78.87556	1068	15	324
10	0.16804	-78.87531	1088	15	454

At each plot, we followed a consistent and standardized protocol of previous surveys of ferns [[6], [7]–8] to evaluate the species richness and abundance. The species richness was recorded as the number of species per plot and the abundance was registered as the number of individuals of each species per plot. For species identification, we collected samples of all species and deposited them at the herbarium HUTI of the Universidad Tecnológica Indoamérica and the herbarium QCA of the Pontificia Universidad Católica del Ecuador. Scientific names were checked at International Plant Names Index (IPNI) https://www.ipni.org [9].

**Fig. 5:**
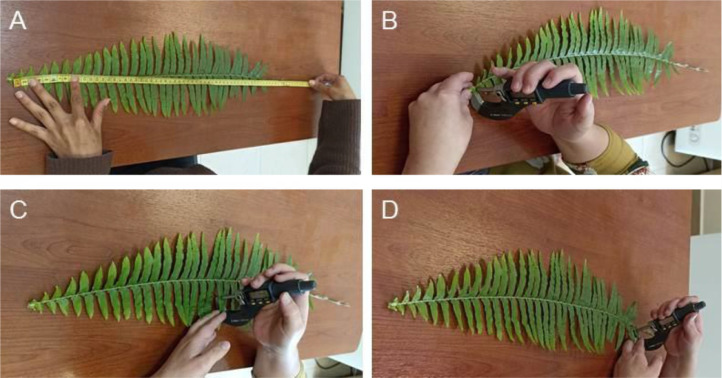
View of the procedure to measure the length (A) and thickness (B-D) of fern leaves.

We measured two plant functional traits of the most representative species per elevational level, the leaf length, and the leaf thickness. For this purpose, we collected leaves from several individuals per species in different quantities outside the plots. We measure the length of fresh leaves from the base to the apex, using a measuring tape (in cm) (Fig. 5A). The leaf thickness was measured also in fresh leaves with a digital micrometer (in µm) at apex, middle and base of each leaf, maintaining a constant pressure and avoiding mid and lateral ribs (Fig. 5B-D) [10].

## Ethics Statements

This work does not involve any type of human studies, animal studies, or data gathered using social media.

This manuscript adheres to ethics in publishing standards.

## CRediT author statement

**Laura Salazar**: Conceptualization, Methodology, Writing - Original draft preparation and writing final manuscript. **Paola Peña:** Data generation, curation. **Renato Segura:** Data generation, curation. **Mateo Roldán:** Writing- Reviewing and Editing.

## Declaration of Competing Interest

The authors declare that they have no known competing financial interests or personal relationships that could have appeared to influence the work reported in this paper.

## Data Availability

Ferns of Mashpi (Original data). (Mendeley Data).
